# Vaccine Depot

**Published:** 1851-01

**Authors:** 


*Vaccine Depot*—We would direct the attention of those of our readers who may be in want of vaccine virus, to the advertisement of Mr. A. I. Mathews, of this city. We are assured by Mr.M., that pains will be taken to obtain supplies from none but reliable sources.

